# Scalable Hierarchical Aggregation and Reduction Protocol (SHARP)^TM^ Streaming-Aggregation Hardware Design and Evaluation

**DOI:** 10.1007/978-3-030-50743-5_3

**Published:** 2020-05-22

**Authors:** Richard L. Graham, Lion Levi, Devendar Burredy, Gil Bloch, Gilad Shainer, David Cho, George Elias, Daniel Klein, Joshua Ladd, Ophir Maor, Ami Marelli, Valentin Petrov, Evyatar Romlet, Yong Qin, Ido Zemah

**Affiliations:** 8 School of Computing, University of Utah, Salt Lake City, UT USA; 9 Cray, a Hewlett Packard Enterprise Company, Seattle, WA USA; 10 Helmholtz-Zentrum Dresden-Rossendorf, Dresden, Germany; 11 Extreme Computing Research Center, King Abdullah University of Science and Technology, Thuwal, Saudi Arabia; 12 Mellanox Technologies, Inc., 350 Oakmead Parkway, Sunnyvale, CA 94085 USA; 13 Mellanox Technologies, Ltd., HaKidma St 26, 2069200 Yokne’am, Israel

**Keywords:** In-network computing, All-reduce, Streaming reduction, Hardware collectives, InfiniBand, Mellanox SHARP

## Abstract

This paper describes the new hardware-based streaming-aggregation capability added to Mellanox’s Scalable Hierarchical Aggregation and Reduction Protocol in its HDR InfiniBand switches. For large messages, this capability is designed to achieve reduction bandwidths similar to those of point-to-point messages of the same size, and complements the latency-optimized low-latency aggregation reduction capabilities, aimed at small data reductions. *MPI_Allreduce()* bandwidth measured on an HDR InfiniBand based system achieves about 95% of network bandwidth. For medium and large data reduction this also improves the reduction bandwidth by a factor of 2–5 relative to host-based (e.g., software-based) reduction algorithms. Using this capability also increased DL-Poly and PyTorch application performance by as much as 4% and 18%, respectively. This paper describes SHARP Streaming-Aggregation hardware architecture and a set of synthetic and application benchmarks used to study this new reduction capability, and the range of data sizes for which Streaming-Aggregation performs better than the low-latency aggregation algorithm.

## Introduction

A parallel application is a collection of independent computational elements that communicate with each other to the degree needed by the application. In tightly coupled High Performance Computing (HPC) applications the type of inter-process communication involved is either some form of point-to-point or collective communication. The Message Passing Interface (MPI) [1] and Open SHMEM [2] define HPC oriented APIs that provide interfaces to such capabilities. Network communication happens between end-points. In point-to-point communication, data is moved from one source to a single destination, and includes operations such as the non-blocking MPI_Isend() and MPI_Irecv() which are used to initiate sending or receiving data, respectively. Collective communication involves some form of data exchange with participation of all members of a group of endpoints, such as MPI_Barrier() which is used to synchronize a set of end-points (MPI processes), or MPI_Allreduce() which is used to gather equal-sized vectors from all members of the collective group, produce a single output vector, and return this to all members of the group.

Collective communication is used by many HPC applications. Efficient implementations of such algorithms often use a chain of point-to-point communication thus serializing algorithm communication, which tends to be scale dependent, with the number of such communication in the critical path increasing with group size. Therefore, collective communication often has a large impact on application scalability.

This scalability challenge has spawned many efforts to optimize collective communication algorithms. Most of these have used host-side logic to manage the collective algorithm as well as the necessary data manipulation with the network being used exclusively as a data pipe. Some network-hardware-based solutions have been implemented, with those relevant to the focus of this paper reviewed in Sect. 2.

Mellanox Technologies, as a provider of HPC network technology, has been moving the implementation of portions of the collective operations to the network, freeing up the computational elements, such as CPUs and GPUs, for computation. For example, CORE-Direct®[10] moved management of the communication dependencies in the chain of collective operations to network hardware in support of asynchronous progress. Mellanox is in the process of IO Processing Units (IPUs) that improve system efficiency by relocating the processing of network operations and data algorithms from the main host into the network fabric. As part of this effort the Mellanox SHARP [9] protocol has been developed to optimize collective reduction and aggregation operations. The first set of capabilities supported include those needed to implement reduction operations, including allreduce, reduce and barrier-synchronization, with a latency-optimized short vector reduction algorithm.

This paper describes and evaluates a new IPU SHARP capability added to Mellanox’s HDR InfiniBand switches. This capability, called Streaming-Aggregation, moves distributed large-data reductions from the host to the network, using a bandwidth-optimized algorithm designed to handle wide radix reduction at near wire speeds. Section 3 describes the Streaming-Aggregation capability introduced in Mellanox’s Quantum^TM^® switch, providing support for long vector reduce, allreduce and broadcast operations. Due to space considerations, we focus only on the reduction operations, and specifically the allreduce operation. Streaming-Aggregation optimizes these often-used global reduction operations by performing the data reduction operations as it traverses a reduction tree in the network. Data from each source is injected into the network only once, and the volume of data is reduced as it goes towards the root of the tree. This is in contrast to CPU-based algorithms where data traverses the network multiple times between network endpoints, to be reduced at each stage at some node in the system. The large-radix reduction trees used provide a highly scalable algorithm and shallow reduction trees, reducing the latency of a one MByte (MB) data reduction across 64 hosts by a factor of 3.5. The effect of this optimization on overall application performance depends on the frequency of using such calls, as well as the skew in the collective initiation across the group of participating processes. The greater the skew, the less pronounced the impact. However, the latter is true for any aggregation algorithm, whether implemented in hardware or software.

Section 2 describes previous work, Sect. 3 describes the Streaming-Aggregation design and Sect. 4 provide some experimental data to demonstrate the effectiveness of this approach in improving system performance, making more CPU cycles available for computation.

## Previous Work

Previous work on reduction algorithms for distributed vectors has included both algorithmic level optimization with software-based implementation as well as work on hardware acceleration of such algorithms. Most of this work is aimed at accelerating small-to-medium data reduction, with relatively little work on optimizing the reduction of longer vectors.

This algorithmic work has resulted in several algorithms in common use today. For long vector reduction Rabensiefner [14] has developed a widely used algorithm baring his name. This algorithm uses a reduce-scatter phase to compute a distributed result vector, with this vector being distributed across members of the communicator, and an allgather step to gather the full vector to all group members. A ring algorithm [14] has also been developed to optimize large vector reductions, and scales linearly with vector size.

Most of the work on hardware optimized collectives has focused on short-vector reduction, with a limited number of published efforts aiming to address large data reduction. The latter faces the challenge of handling very large amounts of data in a single collective operation while staging data across the network for performing the data reduction. In addition, for a given vector length, the total volume of data being reduced, increases with group size, further increasing the amount of data manipulated. To achieve reduction rates similar to the available network bandwidth, such data needs to be reduced efficiently as it is transferred, to form an efficient reduction pipeline. While multiple implementation of short-to-medium vector reductions have been found which offload the full operation to the network, only one reference has been identified on work that offloads the full large-vector reduction. Gao [8] implemented several tree-based reduction algorithms for FPGA-based systems, and ran experiments on a 32 node system. The latencies are reported for messages up to 140 Kbyte (KB) in size are high - on the order of milliseconds. Kumar et al. [11] developed an efficient algorithm for the Blue Gene/Q platform, which leverages the system’s 5D torus with the reductions being performed by the host CPU. Adachi [6] implemented the Rabenseifner algorithm for the K-computer taking advantage its 5D network topology, segmenting the vectors into three parts which are reduced in parallel over three disjoint trees, and using the host CPU to perform the data reductions. Stern [13] developed an FPGA based methodology that is relevant for large reductions, but focuses on small-to-medium reductions.

The methodology being described in this paper offloads the full data reduction operations to the network, with the use of an efficient pipeline to achieve reduction throughput similar to the peak network bandwidth.

## Streaming-Aggregation

Streaming-Aggregation [7] is a new capability introduced with Mellanox’s HDR InfiniBand technology to perform reductions on data in-flight while maintaining near line-rate data transfers. This section describes the hardware enhancements made to the Mellanox SHARP protocol in support of this capability. This new protocol supplements the latency optimized reduction capabilities introduced with Mellanox srp in Switch-IB®-2 EDR switches [9].

**Fig. 1.**
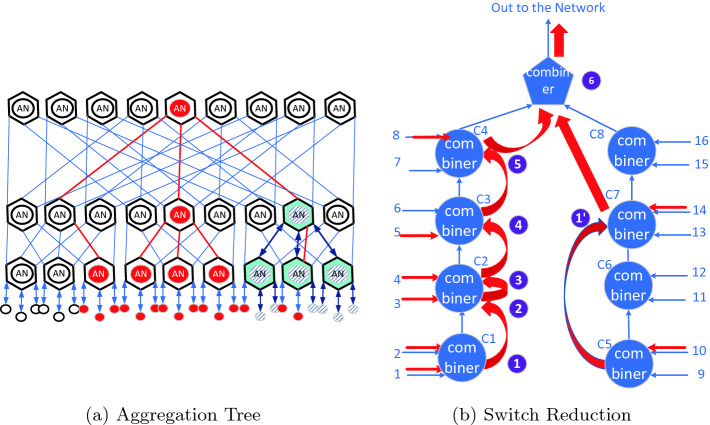
(a) Aggregation tree and a reduction group overlaid on this tree. Switches are displayed as hexagons, nodes as circles and the edges showing switch-to-switch connectivity. (b) Switch-level reduction operation. (Color figure online)

Mellanox SHARP protocol details are described in [9], with a brief summary below. Mellanox SHARP uses reduction trees where the interior nodes of the tree and the root are instantiated in the switches. Hosts serve as the data source and data destination, and are the leaves of the reduction-trees. Figure 1a shows an example of a three-level fat tree and an aggregation group within this tree, with the hexagons representing radix-six switches which include a Collective Functional Unit (CFU) represented by a circle in the switch. Switch connectivity is shown by the edges, and the hosts connecting into the switched fabric by circles. One of several possible reduction-trees that may be defined within this network include the switches with red and cyan aggregation nodes (ANs), with the hosts that are the sources of data colored red or striped. In general, switches need not be ANs within a reduction-tree, as is reflected by the AN in the second level of the red tree on the left hand side, which is not colored.

The AN is used to support Mellanox SHARP’s reduction functionality. These nodes reduce the data received from their children producing one output vector. Interior nodes forward the result to their parent and the root node initiates the result distribution phase, replicating the data to its children. Interior nodes replicate the result received from their parent to their children. The upper limit on the node radix supported by the CFU leads naturally to a hierarchical approach being used to implement collective operations, with a set of levels handling host-side aspects of the collective operations, and the switching infrastructure handling the network-side portion of these collective operations.

Reduction-trees are defined at network initialization time, and reduction groups at run-time. An aggregation-group is defined by the hosts that serve as sources of data for a given set of collective operation. For example, an MPI implementation may create a Mellanox SHARP group at communicator initialization time or on first use. In Fig. 1a the cyan colored nodes and striped hosts define a two-level aggregation-group on the specified reduction-tree.

The following enhancements to Mellanox SHARP have been made in support of the Streaming-Aggregation capability:

- Reduction-trees have a new trait added to specify their type, supporting either low-latency reduction or Streaming-Aggregation.
- The ability to lock a Streaming-Aggregation tree for exclusive use is added. This is done with new capabilities added to the low-latency reduction-tree, with a topology that is identical to that of the Streaming-Aggregation tree.
- Switch-level support for a pipelined reduction ring.
- A single child is supported per tree per switch port. A given aggregation group supports one outstanding operation at a time, with a switch supporting operations on up to two trees at a time.
- A scalable reliable multicast is supported on the tree.

The reduction algorithms are implemented using existing InfiniBand transports, and as such inherit the characteristics of these transports. They include the message size restrictions imposed by InfiniBand and HCA capabilities, such as the gather/scatter capabilities. The low-latency aggregation protocol originally implemented imposes a protocol-specific upper limit, on the order of hundreds of bytes, on the vector size which is well below the InfiniBand message size limit of 2 GB. The Streaming-Aggregation protocol does not impose such additional limitations.

### Tree Type

The Streaming-Aggregation protocol uses a bandwidth-optimized protocol to perform data reductions. A design decision is made to associate a single protocol (e.g., latency-optimized or bandwidth-optimized) with a given reduction-tree, with multiple trees being able to span identical network resources. A protocol trait associated with the tree is used to specify which protocol is supported.

### InfiniBand Transport Selection

To provide an asynchronous aggregation protocol, without requiring host-side intervention, the reduction protocol must provide network-side reliability. In addition, the aggregation protocol is designed to use transport protocols, and not to mix network transport and aggregation elements in a single protocol.

It is desirable to use a reliable transport to send data between nodes towards the root of the reduction-tree and let the hardware transports handle all reliability issues. Such an approach does not slow down the aggregation by waiting on CPU cycles to become available for progressing the protocol, or for timers with long end-to-end timeout periods to expire. The InfiniBand Reliable Connection (RC) transport is favored over the Dynamically Connected (DC) transport because the number of AN-to-AN connections is small, limited by the upper limit on the AN radix, and remains constant unless the network is reconfigured. Therefore, the scalability benefits of DC, with its ability to support multiple destinations are not relevant in this case, which is why InfiniBand RC transport is used for sending data between tree nodes.

Since the result of the aggregation is destined to one or more user-space address spaces, depending on the collective operation being performed, using host-based reliability algorithms as part of an algorithm that handles missing result data is possible. Using Unreliable Datagram (UD) multicast to distribute the results within the tree provides the lowest latency method for distributing the aggregation result within the tree. However, since the protocol is unreliable, a second transmission of the same data is needed, with appropriate handling of duplicate data reception, to ensure that the result is received by each member of the reduction group. For short messages sending the results twice makes sense, once using InfiniBand’s UD multicast transport and then with the reliable RC transport down the tree, as message rate, and not network bandwidth, is the limiting latency factor determining the latency of the result distribution. Duplicate data is handled by receiving data into temporary buffers and copying one result into the user buffer, thus ensuring a second copy is not received into user destination buffers after the user process has already been notified of completion and could be modifying the data. For short messages, the cost of the memory copy is small relative to the overall cost of the UD multicast data distribution, and therefore makes sense from an aggregation latency perspective. However, for large messages, sending the data twice effectively halves the available network bandwidth, doubling the latency, and making such a solution impractical from a performance perspective. Delivering the data to a temporary buffer and the copying it to the user buffer, further increases the cost of distributing the result with UD multicast. Therefore, RC transport is used to distribute the results.

With the aggregation protocols using existing transport protocols, access to these capabilities is through the standard InfiniBand network access mechanisms. Initiating a reduction operation from a given end-point is done by posting a send request. Receive requests for the results are posted to receive queues, InfiniBand completion queues are used to retrieve reduction completion notification. The send request holds an aggregation protocol-specific header as part of the user payload, with the destination address being used to indicate that a message is part of an aggregation operation. New aggregation operations are introduced for the management purposes, such as aggregation-group formation. Space considerations do not allow a discussion of these operations.

### Tree Locking

Streaming-aggregation is designed to perform long-vector distributed data reductions, while maintaining network throughput comparable to that of point-to-point data transfers of the same length. Since data from different children needs to be buffered long enough to combine the data from different sources at a given AN, and there are no guarantees on the temporal nature of data from different sources in specifications like MPI, it is desirable to delay occupying the Streaming-Aggregation buffer resources until all aggregation-group input vectors are ready. This is because the aggregation buffers are a shared switch-level resource that should not be held indefinitely, allowing those operations that are fully ready for the reduction to proceed.

To avoid occupying reduction buffers indefinitely, a protocol for locking a tree for a specified number of streaming-aggregations has been added. This protocol runs on a low-latency aggregation tree with an identical layout to that of the Streaming-Aggregation tree. In addition, the ability to unlock the Streaming-Aggregation tree has been added. This also allows for automatic unlocking of the tree when the number of full message aggregations performed matches the number requested. It is also possible for the tree to be used with no limit on the number of aggregations. This mode is suited for systems that are used to run a single job at time. The mode of operation is set when the lock request is made.

The locking protocol is similar to a Mellanox SHARP barrier operation, with each process in the group initiating a request to lock the tree. These requests propagate up the tree, locking the resources along the way. In the event that a resource is already locked and is unavailable, the failed request is propagated up the tree, with the root sending a failed-lock notification down the tree causing locked resources to be released, and the calling host process to be notified of the failure. The cost of such a lock is similar to a barrier-synchronization operation on the same low-latency aggregation tree.

As noted above, for resource locking purposes, each reduction-tree is associated with a low-latency reduction-tree of identical layout.

### Reduction Tree

The Streaming-Aggregation reduction-tree is very similar in nature to the low-latency reduction-trees, with respect to how the aggregation proceeds. Individual ANs receive data from a predetermined number of children and reduce the data to produce a single output vector. Interior aggregation-group nodes forward the data to their parent, and the root of the aggregation-group initiates result distribution. An important feature of the aggregation protocol is that a single result is forwarded towards the root of the tree from each AN thereby reducing the amount of data forwarded by its aggregation radix. Similarly, as data is distributed from the root, it is replicated once per child at each AN, keeping the volume of data transferred to a minimum, and generally transferring much less data than that of host-based algorithms.

### Reduction Pipelining

To achieve high network bandwidth with long vectors while performing a data reduction, an efficient pipeline needs to be established, which supports data staging into the arithmetic units. These units are then used to operate on the data, while maintaining high end-to-end data throughput. This data motion must be maintained throughout the full distributed reduction data path.

To achieve good pipelining InfiniBand’s credit-based mechanism is used as a means for the responder (e.g., the AN) to inform the producer, i.e., the source of the data, of its available buffer space. This allows data to be sent between the two at an optimal rate, while avoiding overwhelming the responder with data it must drop. The credit mechanism runs over a reliable InfiniBand transport. This synchronizes the responder and requester by sending the amount of credits in response packets from the responder and allowing sending an additional single “limited” packet when the requester runs out of credits. This is as described in the InfiniBand specification for handling end-to-end credits.

### Switch-Level Reduction

The AN in each switch takes input from a pre-configured set of children, and then delivers the data to the pre-configured destination, as shown in Fig. 1b. There is a one-to-one mapping between children and physical ports, on a per reduction-tree basis. Switch ports are paired and assigned reduction resources. The ports are arranged in two half rings, which meet in the middle, with the top CFU producing the final result and sending it to the destination. Data is supplied to the reduction tree at MTU granularity, which enables setting up an efficient pipeline capable of achieving end-to-end reduction at near wire speed.

Figure 1b shows how a switch performs the reduction of data coming from eight sources, for a switch of radix 16. The red arrows represent the children for the reduction at the AN, with the circles representing the Streaming-Aggregation reduction unit that handles data from two ports, and the pentagon represents the CFU which produces the final result. On the left-hand branch there are five reduction steps, with the first reduction taking data from the bottom two ports forwarding the result to the second combiner.

On the left-hand side, data from ports 1 and 2 are combined by C1. The result is forwarded to C2 where it is combined with the input from port 3 with the result being combined by C2 with the input from port 4, and forwarded to C3. At C3, the forwarded data is combined with the data from port 5 and forwarded to C4, where the data is combined with the data from port 8.

On the right-hand side, data from port 10 is forwarded through the combiner in C6 to C7 where it is combined with the data from port 14. The result is forwarded through C8 to the CFU where it is combined with the data from C4, and sent out to the appropriate exit port to the next AN in the tree.

At each step through the switch, data is processed at near wire speed, providing good throughput, with sufficient switch resources to keep the pipeline busy, supporting near full wire speed reduction.

### Result Distribution

With the reduction complete at the root of the aggregation-group, it is ready to be distributed to data recipients, be it the host in the group for an allreduce type of operation, or the root of a reduction operation. With the short message, the latency-optimized hardware multicast protocol is used to provide low-latency data distribution, and a reliable transport is used to send the result down the tree to ensure reliable data distribution. For a bandwidth-oriented protocol, distributing the result twice, with both reliable connections and UD multicast protocols, reduces the operations bandwidth by a factor of two, making it a poor option.

**Fig. 2.**
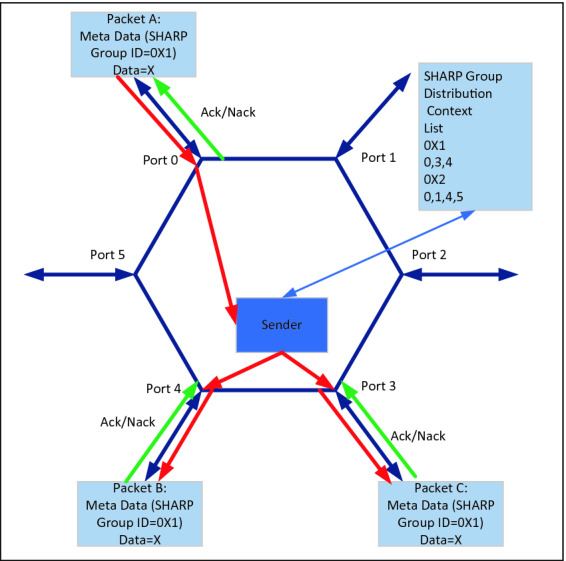
Reliable Data Distribution. Black arrows represent ports, red arrows represent the data path and green arrow represent the control path. (Color figure online)

Therefore, a new reliable broadcast protocol has been developed to distribute the data reliably at near wire bandwidths. This protocol uses unicast messages to send data between nodes in the aggregation-group, encapsulating the Mellanox SHARP reduction-tree which is used to distribute the data. When the CFU receives the unicast message, it extracts the SHARP group handle, and uses this to look up in its local SHARP group tables the list of ports through which the data needs to be forwarded. An optimized reliable packet generator is used to replicate the data which is sent out through each of the ports constructing new RC messages, one for each of the group destinations. This is depicted in Fig. 2 for a radix-6 switch and SHARP group 0X1. The process of extracting the SHARP group handle, replicating the reliable packets and sending the data to the next nodes in the reduction-tree is continued until the data reaches the destination.

### Aggregation Protocol Resilience

The high performance computing community has yet to converge on a set of agreed upon protocols to handle application-side error recovery. Therefore, the protocol is designed to allow users to select their own method of handling aggregation protocol failure.

With network error rates being rather low, with the average duration between unrecoverable errors being orders of magnitude higher than that of the longest aggregation protocol duration, Mellanox SHARP’s mode of handling errors is limited to notifying the data sources when failure occurs, and letting the user decide how to proceed. Upon failure, the affected aggregation trees are torn down, and it is up to the host-side SHARP stack to decide if to re-initialize the SHARP resources, with a potentially new network configuration (i.e., without the failed resources).

Once the running application receives notification that a given aggregation has failed, it can decide how to proceed. It can try and re-initialize the application SHARP resources and use them again, or use an alternative host-based algorithm, which bypasses the affected resources, and restarts the affected aggregations. In addition, since successful local aggregation protocol completion does not imply success across the full reduction group, the application is free to add an agreement protocol, with the associated costs, before declaring the operation complete and returning control over the result buffers to the user.

## Experiments

The Mellanox SHARP Streaming-Aggregation capability is studied using synthetic benchmarks and full applications, to explore the performance characteristics of this capability and its impact on applications.

### Test System Configuration

The primary system used to run the synthetic benchmarks included 64 nodes of dual 18 core sockets of Intel(®) Xeon(®) Gold 6154 CPU running at 3.00 GHz. Each host uses the Red-Hat Linux version eight package and MLNX_OFED_LINUX-4.7-1.0.0.1. Each node is connected to network using a ConnectX®-6 HDR InfiniBand Mellanox HCA which were connected to HDR InfiniBand Mellanox Quantum^TM^ switches. Each host is able to send data at the limit imposed by the PCIe Gen-3.0x16 bus, which is just above 100 Gbit/sec. The switches are connected in a two-level fat-tree topology, with four InfiniBand HDR Quantum^TM^ L1 switches connected to one Quantum^TM^ L2 switch. The HCAs used firmware version 20.26.1040, and the switches used firmware version 27.2000.2306.

The single switch scalability tests were run on a 32-node cluster. It has 16-core dual-socket Intel(®) Xeon(®) CPU E5-2697A v4 (Broadwell) running at 2.60 GHz with 256 GB of physical memory. Operating system is CentOS 7.7.1908 with kernel version 3.10.0-1062.4.1.el7.x86_64 and MLNX_OFED 4.7-1.0.0.1. Cluster nodes are connected with ConnectX-6 HDR100 InfiniBand and a Quantum^TM^ switch. DL-Poly was also run on this system.

In addition, an 8-node cluster with AMD EPYC 7742 64-core Processors with MLNX OFED version 4.7 running the RDY1003B BIOS connected to a ConnectX®-6 HDR InfiniBand HCA via a PCIe Gen-4 bus was also used to measure performance on a fully enabled single-stream HDR configuration. Availability of systems with PCIe Gen-4 based CPUs has limited most of the testing to network injection bandwidths limited to just over 100 Gbit/s.

The MLPerf data was collected on an HPE Apollo 6500 configured with 8 NVIDIA Tesla V100 SXM2 with 16 GB of memory. The CPU used was a dual socket HPE DL360 Gen10 Intel Xeon-Gold 6134 (3.2 GHz/8-core/130 W) running Ubuntu 16.04, and connected via a PCIe Gen-3 PCI bus to HDR100 HCA running at 100 Gbit/sec connected to a single Mellanox Quantum^TM^ switch.

The MPI from HPC-X version 2.5 [3] was used in the experiments.

### Synthetic Benchmarks

The OSU allreduce [4] benchmark is used to study the performance of reduction capability. The test is modified to report the achieved bandwidth, in addition to the latency, where the bandwidth is computed as the message size divided by the measured latency. This is done to assess the hardware’s ability to utilize available network bandwidth while performing the data reduction.

Measurements were taken to assess the efficiency at utilizing available network bandwidth, its efficiency compared to the low-latency aggregation capability and host-based distributed reduction algorithms. The host-based algorithm used is a radix-2 Rabenseifner’s algorithm [14] - reduce-scatter followed by an allgather. In addition, Streaming-Aggregation’s performance as a function of switch configuration and job size is studied. All the experiments described in this subsection focus on the in-network Streaming-Aggregation feature, so only a single process is used on each host.

**Fig. 3.**
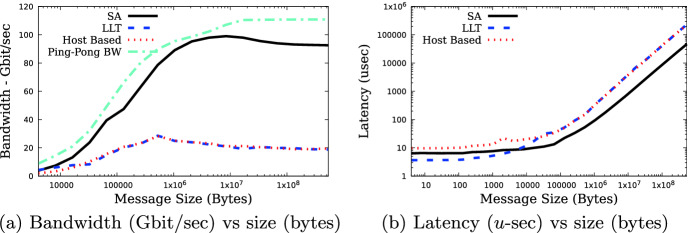
(a) Streaming-aggregation (SA), low-latency aggregation (LLA), host-based MPI_Allerduce implementations and MPI ping-pong bandwidth. (b) Streaming-aggregation (SA), low-latency aggregation (LLA) and host-based MPI_Allreduce.

The efficiency of the Streaming-Aggregation and its performance relative to the low-latency aggregation and host-based implementations was measured using all 64 hosts, with 16 hosts attached to each leaf switch. Ping-pong bandwidths are also reported. The results of these experiments are displayed in Fig. 3a. As this figure shows, the allreduce bandwidths obtained by the Streaming-Aggregation are close to that obtained in the ping-pong experiment which transfers data without manipulating it. The bandwidths obtained are much higher than those obtained with the host-based reduction operations, varying from a factor of about 2 higher at 4 KB message size to a factor of 4.8 higher at 256 MB message size. The bandwidth obtained is also higher than that obtained with the low-latency aggregation protocol, being similar at 8 KB message size and similar to the host-based performance at large message size. The reduction bandwidth achieved peaks out at about 96% of the ping-pong bandwidth, dropping off a bit at larger message sizes.

The Streaming-Aggregation is designed for long message aggregation, whereas the low-latency aggregation is designed to optimize for the small data reductions, which are dominated by latency effects. It is therefore important to figure out at what message size to switch from using the low-latency aggregation to the Streaming-Aggregation algorithm. Figure 3b compares the MPI_Allreduce latency obtained using Streaming-Aggregation, low-latency aggregation and the host-based algorithm. As expected, the hardware offloaded latency is better than that of the host-based algorithm, with the latency optimized algorithm performing better at small message sizes, and bandwidth optimized algorithm overtaking it in the range of 4 to 8 KB. These measurements do not take into account the cost of reserving the Streaming-Aggregation resources, for those instances in which this reservation is required. In such instances, the cross-over point will be at a larger message size. The overheads of managing multiple message data segments in the reduction pipeline, includes a credit mechanism, as well data orchestration logic within the AN, which is absent from the low-latency aggregation protocol. It is such logic that enables the high-bandwidths supported by the Streaming-Aggregation protocol, but increases the latency, and is independent of the data source.

**Fig. 4.**
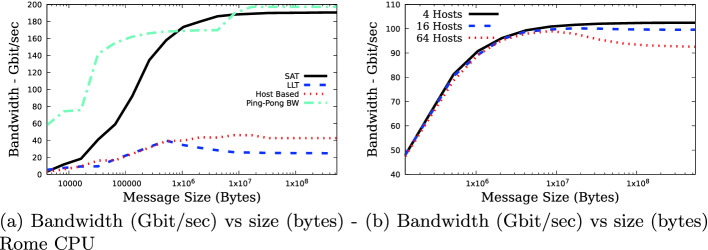
(a) Streaming-aggregation (SA), low-latency aggregation (LLA), host-based MPI_Allerduce implementations and MPI ping-pong bandwidth - Rome CPU. (b) MPI_Allreduce Streaming-aggregation using four leaf switches and varying the number of hosts per switch.

Bandwidth was also measured on an AMD Rome cluster, supporting a PCIe Gen-4 bus, which enables full HDR throughput. The reduction bandwidth as a function of message size is displayed in Fig. 4a, peaking at close to 95% of available network bandwidth and 96% of ping-pong bandwidth, which is about 4.5 times that of the host-based algorithms, and about a factor of 7.6 better than using the low-latency aggregation capabilities.

Several other comparisons are made to further study the behavior of the Streaming-Aggregation capabilities. Single switch measurements were performed to understand how the distributing the reduction between the two reduction rings in a single switch impact performance. Since the setup available had 32 nodes per switch, 16 hosts *MPI_Allreduce()* runs were made varying the configuration from all 16 nodes on a single ring, to half and half. As expected, this showed no discernible impact on the MPI-level reduction latency and bandwidth.

**Fig. 5.**
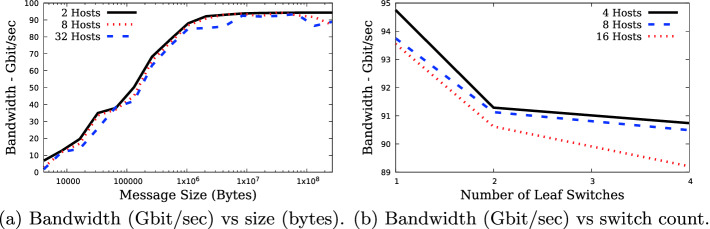
(a) Single switch MPI_Allreduce Streaming-aggregation reduction bandwidth (Gbit/sec) as a function of message size. (b) 1048576 byte message size MPI_Allreduce bandwidth (Gbit/sec) as a function number of hosts per switch, for a fixed number of total hosts. The number of switches varies from one to four.

Figure 5a shows the *MPI_Allreduce()* bandwidth as a function of message size with all the hosts connected to the same switch and a variable number of hosts. As this figure shows, the host count has a very small impact on the measured reduction bandwidth.

Figure 4b shows the *MPI_Allreduce()* bandwidth as a function of message size and the number of hosts per switch, for a 4 switch two-level fat-tree configuration. For this particular configuration the number of hosts has minimal impact on measured bandwidth up to about 4 MB message size, but with 16 hosts per switch we see a drop of about 7% in measured bandwidth. Host based measurements show a corresponding drop of about 12%.

Figure 5b showed the *MPI_Allreduce()* bandwidth for fixed total host count and an increasing number of leaf switches, decreasing the number of hosts per switch with increased switch count. Increasing the total number of hosts has only a small impact on overall bandwidth, with the largest impact being on the case where 16 hosts are in use dropping by about 4.8% going from one to four switches. The drop from two to four switches (both require using both levels of the two-level fat-tree) is only about 1.6%. The corresponding drops in performance for the host-base algorithm are 1.9% and 0.3%.

**Fig. 6.**
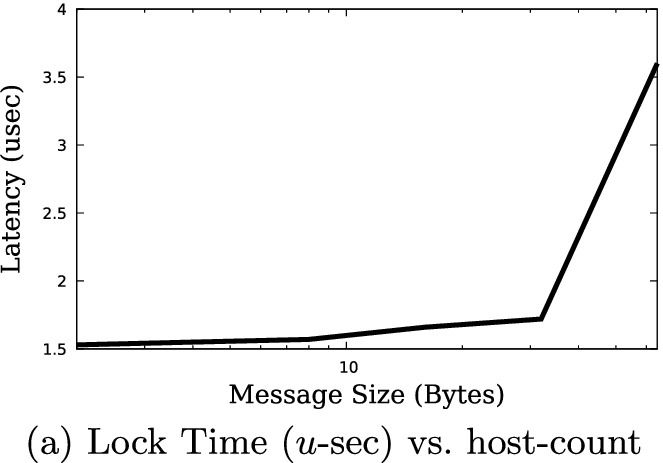
(a) Streaming-Aggregation tree lock time, as a function of host count. 2 to 32 nodes are attached to a single switch and 64 hosts are configured in a two level fat-tree.

Locking the tree for the aggregation operation can be done either for the duration of the life of the application, such as the lifetime of an MPI communicator, or for a specified number of reduction operations, thus allowing other reduction trees to use the resources. Figure 6a shows the latency of the reduction operation in the range of 2 to 64 hosts. The range of data points between 2 and 32 hosts was measured on the single switch Intel based system, and the 64-node data on the two level fat-tree configuration. The cost of the lock is indeed similar to that of the barrier operation, with a very small increase in latency for the fixed single-switch configuration and the expected increase in latency going from one to two levels in the reduction tree.

### Application Benchmarks

The impact of the Streaming-Aggregation on the performance of two applications, DL-Poly and PyTorch is studied. These are described below.

**DL-Poly** [15] is a classical molecular dynamics code developed at Daresbury Laboratory. The bars in Fig. a show the total run-time of the Sodium Chloride melt with Ewald sum electrostatics and 27 K atoms (bench4) as a function of host count, with (orange bars) and without (blue bars) using the Streaming-Aggregation. The line plot represents the overall improvement in application run-time as a percent of total application run time. Measurements were taking using a host count varying between 2 and 24, with 32 processes per node. The amount of time spent in the large $MPI\_Allreduce()$ operations at 24 nodes and 32 ranks per node is 6.85 s out of a total run time of 45.02 s, or about 15%. The Streaming-Aggregation reduces this time to 4.73 s, and is used to reduce vectors of size 524288, 196608 and 98304 bytes. As the results indicate, using the Streaming-Aggregation capabilities to speedup the *MPI_Allreduce()* operations improved overall simulation time by as much as 4% at 22 nodes, and about 2.5% at 24 nodes. Reduction costs at these different sizes are similar, with most of the fluctuations in run-time coming from other parts of the code.

**Fig. 7.**
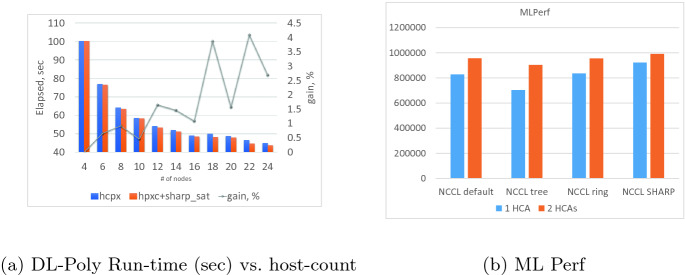
(a) DL-Poly run time (sec) as a function of host count, with 32 process per host using Streaming-Aggregation.Test case: Bench4 - Sodium Chloride, 27 K atoms. (b) MLPerf performance using PyTorch (Color figure online)

**PyTorch** [12] is a machine learning library used in computer vision and natural language processing. This was used to run the Transformer Translation model [16] MPLerf benchmark, with and without using the Streaming-Aggregation capabilities. The performance on a 4 host 8 GPU system, using one and two HDR100 interfaces is shown in Fig. 7b, with the data being reduced from the GPU buffers. The reduction capabilities are exposed through the NVIDIA Collective Communication Library (NCCL) [5] which also includes support for Mellanox’s Streaming-Aggregation capabilities. As the figure shows, using the Streaming-Aggregation capabilities improves the benchmark performance relative to the default tree and ring reduction algorithms used by NCCL. The single HCA performance is improved by about 10% relative to the ring-based reduction algorithm, with the two-HCA performance improving by 3.7%. Incidentally, the GNMT MLPerf benchmark running on 24 DGX1V nodes and the VAE benchmark running on 32 DGX1V nodes, using 4 parallel HDR networks and enabling the Streaming-Aggregation improves performance by 18% in both cases, but analyzing these is beyond the scope of this paper. The vectors being reduced are long, as shown in Table 1. When NCCL’s ring algorithm is used for the reduction 28% of total run time is spent in reduction, but when Mellanox SHARP is used this drops to 20% of total run time.

**Table 1.** MLPerf transformer translation model reduction message distribution. The data type is 16 bit floating point.

# Calls	Message count	Message size (MByte)	# Calls	Message count	Message size (MByte)
1	210808832	402	1100	46169088	88
2200	46171136	88	1100	72297472	137

As Mellanox SHARP Streaming-Aggregation performance optimization efforts continue, we expect to improve the performance of the aggregation operations. Improvement in application performance will depend on how this capability is used.

## Summary

This paper describes the Mellanox SHARP Streaming-Aggregation capability introduced in Mellanox’s HDR InfiniBand network hardware. It takes in vectors from different network end-points, reduces the data to produce a single output vector, which is then distributed to the specified nodes in the network. No software is used in the reduction path.

As the *MPI_Allreduce()* OSU benchmark results show, the efficiency of the data reduction and distribution is close to that of the point-to-point bandwidth, achieving good pipeline efficiency in reducing and forwarding data. On a 64-node HDR system using a PCIe Gen-3 bus to connect to the network a reduction efficiency of as high as 96% of ping-pong message efficiency for a 2 MByte message, and at 64 KB achieves about 80% efficiency. Peak reduction bandwidth is achieved with messages of size 8 MB. When the bandwidth limitation imposed by the PCI bus is removed, using a PCIe Gen-4 bus, the bandwidth reaches 59 Gbps ate 64 Kbyte, which is 3.5 times higher than with the host-based algorithm. With 67 GB message size it peaks at 190 Gbps, which is 95% of the network bandwidth and 4.45 times higher than with the host-based algorithm.

Comparing Mellanox SHARP Streaming-Aggregation bandwidth to that obtained using a host-based approach, a large increase in measured bandwidth using the new capabilities is observed. As the data from Fig. 3a shows, Streaming-Aggregation bandwidth is about a factor of two higher than the host-based reduction algorithm bandwidth at 4 KB message size, and close to a factor of five greater for messages of size 128 KB and above. Similarly, the Streaming-Aggregation reduction bandwidth is greater than the low-latency aggregation based reduction bandwidth, for all but the 4 KB message size.

For small message sizes, the latency of the low-latency aggregation based reductions is lower than the Streaming-Aggregation based algorithm, with both being lower than the host-based algorithm. For the 64 host configuration the cross-over point between the algorithms is between 4 and 8 KB, and when tree locking is necessary, this increases to about 16 KB. The cost of managing and pipelining multiple data segments with the Streaming-Aggregation is what makes the short message aggregation less efficient then when using low-latency aggregation. When more than two to three message segments are required using the low-latency aggregation protocol it is more efficient to use the Mellanox Streaming-Aggregation protocol.

As a basic capability, the addition of the Streaming-Aggregation functionality enables the asynchronous offloaded reduction capabilities to supersede the host-based algorithms. Also, using these capabilities with DL-Poly and PyTorch shows this to be a viable alternative to host-based reduction algorithms at the full application level, improving the application performance for the tests run by up to 7% and 10% for DL-Poly and PyTorch, respectively.

Finally, studying Streaming-Aggregation as a function of network configuration has shown that performance remains as the system size increases, albeit with some reduction in bandwidth. This is expected with a longer data path which increases the latency for the first MTU to reach the destination, thus reducing the measured bandwidth. Factors that are local to a single switch have a much smaller impact on performance relative to factors such as reduction tree depth and the width of the reduction tree. The distribution of hosts across the reduction rings in the switch had no discernible effect on the end-to-end reduction performance, while the number of hosts per switch was shown to have a small effect. The largest measured impact seems to be related to the reduction tree depth, with a smaller impact exerted by the number of switches used at a given tree depth. As larger switch configurations become available for testing, the impact of scale on overall measured bandwidth can continue to be studied. The large-radix reduction supports shallow reduction trees, with a three level tree able to support systems with over 10,000 nodes using 40 port switches as building blocks.

To summarize, the Streaming-Aggregation capability has been shown to significantly improve the distributed reduction performance of medium and large messages relative to both low-latency aggregation hardware Mellanox SHARP and host-based software reduction implementations. It provides reduction throughput similar to that of point-to-point traffic, and improves the performance of both synthetic and full applications.
